# Gasdermin D: A New Inflammatory Biomarker in Assessing Clinical Disease Activity in Crohn’s Disease

**DOI:** 10.5152/tjg.2025.24726

**Published:** 2025-05-20

**Authors:** Osman Cagin Buldukoglu, Serkan Ocal, Galip Egemen Atar, Besir Kaya, Muhammet Devran Isik, Gul Aydin Tigli, Serdar Akca, Ferda Akbay Harmandar, Yesim Cekin, Ayhan Hilmi Cekin

**Affiliations:** 1 Department of Gastroenterology, Antalya Training and Research Hospital, Antalya, Türkiye; 2 Department of Medical Microbiology, Antalya Training and Research Hospital, Antalya, Türkiye

**Keywords:** Crohn’s disease, gasdermin D, outcome, pyroptosis, severity

## Abstract

**Background/Aims:**

Crohn’s disease (CD) is an inflammatory, progressive disorder requiring monitoring of treatment response and disease course. Gasdermin D (GSDMD), a protein belonging to the gasdermin protein family, plays a role in inflammatory cell death, and activation of GSDMD has been shown to be a component of the pathogenesis of inflammatory bowel disease.

Considering the role that GSDMD plays in inflammation, it was hypothesized that disease activity in CD may be correlated with serum GSDMD levels. The aim of this study was to assess the strength of GSDMD in predicting clinical disease activity in patients with CD in a prompt and easy manner.

**Materials and Methods:**

This cross-sectional study was conducted over a span of 22 months from September 2022 to June 2024. A total of 61 patients with CD were included in the study. Demographic data, disease- and treatment-related data, and laboratory workups of the patients were recorded and analyzed.

**Results:**

Gasdermin D levels were statistically significant in their correlation with the Harvey–Bradshaw Index (HBI) scores of the study population (*P* = .019). A threshold value of 5 ng/mL for GSDMD had a sensitivity of 84.6% and a specificity of 91.7% in differentiating patients with remission or mild disease from those with moderate or severe disease, according to HBI.

**Conclusion:**

This pioneering study revealed that serum GSDMD can be used as a biomarker to assess clinical disease activity in patients with CD. Future studies incorporating colonoscopic evaluation into the equation will provide more insight into the use of this protein as a surrogate marker of disease progression in CD.

**Main Points**

- Crohn’s disease is a chronic, debilitating disorder that requires close monitoring.
- Several biomarkers are commonly used in clinical practice to assess disease activity, with more being investigated.
- Gasdermin D is a protein involved in inflammatory pathways, and studies have shown its role in the pathogenesis of inflammatory bowel disease.
- Results of this study revealed that serum Gasdermin D levels are correlated with clinical disease activity in patients with Crohn’s disease; thus, they can be used as a biomarker to track disease course and/or treatment response.

## Introduction

Crohn’s disease (CD) is an inflammatory bowel disease with a progressive and destructive nature. The disease can affect any part of the gastrointestinal tract. Most cases are diagnosed at a young age; thus, early diagnosis and prompt initiation of treatment are crucial for both achieving disease remission and preventing disease-related complications. Crohn’s disease pathogenesis is complex and multifactorial, with genetic predisposition, alterations in the immune system, and environmental factors playing a part in the process.^1^ Although new treatment options have recently become available and are still being developed in the form of small-molecule therapies and biologics, a significant portion of patients with CD still require surgical therapeutic interventions during their disease course.^2^

Monitoring treatment response and assessing disease activity are the cornerstones of patient management in CD. Since mucosal healing is a key treatment target, colonoscopy is the mainstay modality for both diagnosis and follow-up of the disease.^3^ However, colonoscopic evaluation can be burdensome for the patient and/or the healthcare team, and the procedure-related risks can be a limiting factor in daily practice. Several biomarkers are being used to predict disease activity and outcomes such as white blood cell (WBC) count, erythrocyte sedimentation rate (ESR), and C-reactive protein (CRP). Additionally, new promising molecules and genetic polymorphisms are being investigated.^4^

Gasdermin D (GSDMD), a protein belonging to the gasdermin protein family, plays a role in inflammatory cell death, and activation of GSDMD has been shown to be a component of inflammatory bowel disease pathogenesis.^5^

Considering the role that GSDMD plays in inflammation, it was hypothesized that disease activity in CD may be correlated with serum GSDMD levels. The aim of this study was to assess the strength of GSDMD in predicting clinical disease activity in patients with CD in a prompt and easy manner.

## Materials and Methods

### Study Design and Patient Selection

This cross-sectional study was conducted at Antalya Training and Research Hospital, Antalya, over a span of 22 months, from September 2022 to June 2024. Patients with Crohn’s disease diagnosed at Antalya Training and Research Hospital and who were on follow-up at the inflammatory bowel disease (IBD) outpatient clinic of Antalya Training and Research Hospital were enrolled in the study. Patients without outpatient clinic follow-ups, those with missing records, or patients who did not give consent to participate in the study were excluded. Blood samples for GSDMD were obtained from participants at the same blood sampling session for serum parameters used in the study. Patients with active perianal disease, fistulas, or abscesses were excluded from the study, as these inflammatory processes would potentially alter the study results, regardless of disease activation. Patients with a concurrent infection of any kind, malignancy, and inflammatory-driven disorders were not enrolled in the study, as these diseases would alter the inflammatory status of the patient and thus serum GSDMD levels. Overall, 61 patients were enrolled in the study.

Age, body mass index (BMI), sex, smoking status, time in months since CD diagnosis, treatment the patients were on at the time of enrollment in the study, concurrent use of steroids, previous treatment history for CD (medical and surgical), CD involvement site (ileum-only versus ileocolonic involvement), presence of extraintestinal manifestations (EIMs), and Harvey–Bradshaw Index (HBI) scores were collected and recorded on the day of GSDMD blood sampling. Laboratory work performed in the study comprised CRP, ESR, WBC, and GSDMD.

Patients enrolled in the study were divided into 2 groups in terms of the extent of disease involvement: disease involvement limited to the ileum (ileum only) and ileocolonic involvement. Patients were also split into 2 groups according to the treatment they were on at the time of enrollment. The first group comprised patients on conventional therapy, which included azathioprine treatment with or without 5-aminosalicylic acid. The second group comprised patients on biologic therapy, which included biological agents and small-molecule therapies. Concurrent corticosteroid usage was also recorded and analyzed, regardless of the dosage and type of corticosteroids.

The Harvey–Bradshaw Index is an index used for the stratification of the severity of CD. The index consists of questions regarding general well-being, number of liquid or soft stools, abdominal pain, the presence of an abdominal mass, and disease-related complications including arthralgia, erythema nodosum, uveitis, aphthous ulcers, pyoderma gangrenosum, anal fissures, abscesses, and new fistulas. A point is allocated to each parameter, and the total score is calculated. A score below 5 is accepted as remission, whereas scores 5-7, 8-16, and >16 correspond to mild, moderate, and severe disease, respectively.^6^

### Analysis of Serum Gasdermin D Levels

Serum GSDMD levels were measured using Human Gasdermin D enzyme-linked immunosorbent assay kits (Sunredbio, China). Blood samples of 5 mL were obtained from each patient enrolled in the study. The blood samples were centrifuged at 4000 revolutions per minute for 10 minutes and cold-stored at −80°C until the day of laboratory workup. The results were expressed in ng/mL.

### Statistical Analysis

Categorical variables were presented with frequencies (n) and percentages (%), and continuous variables were presented as the mean (± SD) or median with minimum and maximum values. The Shapiro–Wilk test was used to evaluate whether the data set was normally distributed. Fisher’s exact test was performed to determine the relationship between categorical variables. Mann–Whitney *U* and Kruskal–Wallis tests were performed for non-parametric comparisons of continuous data. Linear regression analysis was performed to analyze the effects of independent variables on GSDMD as well as on HBI. Variables with a *P* value below .1 were included in the multivariate regression analysis. The level of significance was *P* < .05. All statistical analyses were performed using SPSS version 25.0 for Windows (IBM SPSS Corp.; Armonk, NY, USA).

### Ethics Approval and Consent to Participate

This research was approved by the Ethics Committee of Antalya Training and Research Hospital (Date: August 11, 2022, No: 2022-243). The study was conducted in accordance with the Declaration of Helsinki. Written informed consent was taken from each participant in the study. Approval was received from all participants to publish the article.

## Results

### Demographics and Study Parameters of Patient Population

Overall, 61 patients with CD were enrolled. Age, time in months since CD diagnosis, CRP, ESR, WBC and GSDMD levels, and HBI scores of the study population did not have a normal distribution, so these results are presented as median (with minimum and maximum values), whereas other data are presented as mean ± SD.

Twenty patients (32.8%) were female. The median age of the patients was 40 (21-80) years. The mean BMI of the study population was 23.0 (±2.4) kg/m^2^. Eight patients (13.1%) were active smokers. The median disease duration time was 49 months (6-241). The median WBC count of the study population was 7900/mm^3^ (1300-14 700). The median ESR and CRP levels of the study population were 12 mm/h (1-63) and 6.0 mg/L (0.1-53.5), respectively. The median GSDMD level of the patients was 3.08 ng/mL (0.88-12).

More than half of the patients were on conventional therapy (n = 35, 57.4%). Three patients (4.9%) were using concurrent corticosteroids as adjuncts to their therapy. Twelve patients (19.7%) had prior surgery for CD. Thirty-one patients had disease involvement in the ileum only (50.8%), and 30 patients had ileocolonic involvement (49.2%). Thirteen patients (21.3%) had EIM, which comprised joint (n = 6, 9.8%), skin (n = 5, 8.1%), and ocular manifestations (n = 2, 3.2%). According to HBI, 30 patients were in remission (49.2%), 18 had mild disease (29.5%), 12 had moderate disease (19.7%), and 1 patient had severe disease (1.6%). The median HBI score of the study population was 5 (1-17).

Characteristics and laboratory data of the study population are given in Tables 1 and 2.

### Relationship of Gasdermin D and Other Study Parameters with Harvey–Bradshaw Index

The indicator of clinical disease activity for CD used in the study was HBI. The relationship of study parameters with HBI was investigated.

**Figure 1.**
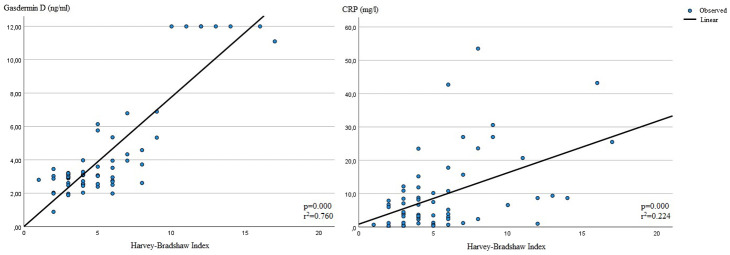
Relationship of serum Gasdermin D and C-reactive protein levels with Harvey–Bradshaw Index (line graph).

The age and sex of the patients were not found to be correlated with HBI. Patients with a lower BMI had higher HBI scores (*P* = .006). Treatment modality and concurrent steroid use were not associated with disease activity according to HBI. The duration of disease, disease involvement site, previous surgery, and presence of EIM were also not found to be correlated with HBI scores of the patient population. White blood cell count and ESR levels were not related to HBI, whereas CRP and GSDMD levels were strongly correlated with HBI scores of the study population (*P* = .000 for both parameters, Figure 1). Patients in clinical remission according to HBI had a median GSDMD level of 2.88 (0.88-3.97), whereas patients with mild disease, moderate disease, and severe disease had median GSDMD levels of 3.29 (1.98-6.79), 12 (2.61-12), and 11.1, respectively. Smokers also had higher HBI scores, and this correlation was statistically significant (*P* = .008).

The relationship of independent variables with HBI was investigated using multivariate logistic regression analysis. The results of the multivariate logistic regression analysis revealed that serum CRP and GSDMD levels were independently correlated with higher scores of HBI (*P* = .024, Odds ratio [OR]: 1.126 and *P* = .019, OR: 2.595). The results of the multivariate logistic regression analysis are given in Table 3.

**Figure 2.**
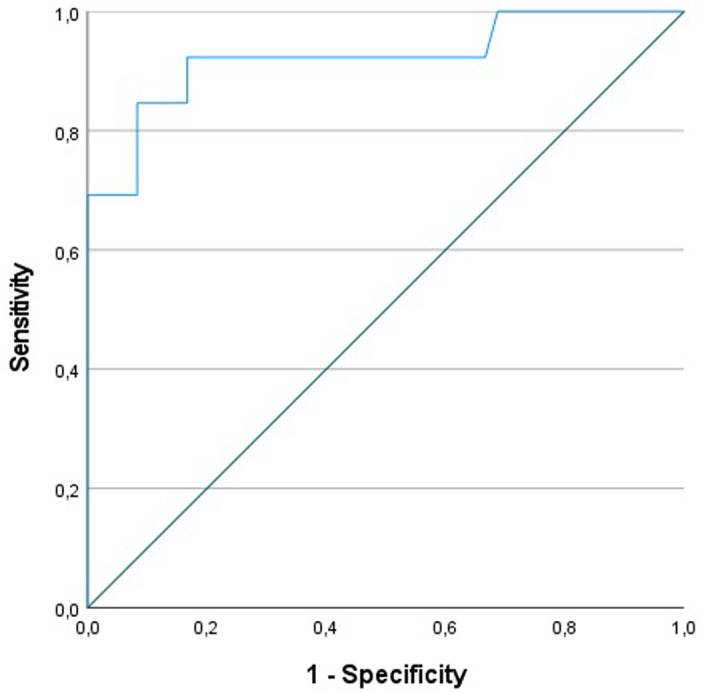
Receiver operating characteristic analysis between Gasdermin D level and Harvey–Bradshaw Index.

Receiver operating characteristic (ROC) curve analysis was performed to assess the strength of GSDMD in predicting clinical disease activity in CD in terms of HBI. The study population was split into 2 groups in terms of HBI for analysis purposes. Patients in remission (HBI score below 5) and patients with mild disease (HBI scores from 5 to 7) were designated as group 1, whereas patients with moderate disease (HBI scores from 8 to 16) and 1 patient with severe disease (HBI score above 16) were designated as group 2. Receiver operating characteristic curve analysis was performed, and an area under the ROC curve value of 0.92 was calculated, revealing the potency of GSDMD in predicting a more severe disease state in terms of HBI. Receiver operating characteristic curve analysis is given in Figure 2.

Following ROC analysis, the study data were evaluated with the aim of determining a threshold level for GSDMD in predicting clinical disease activity in terms of HBI. Utilizing the aforementioned 2 patient groups, a cutoff value of 5 ng/mL for GSDMD had a sensitivity of 84.6%, specificity of 91.7%, positive predictive value of 73.3%, and negative predictive value of 95.6% in predicting more severe clinical disease activity according to HBI.

Since GSDMD is an inflammatory biomarker, it was hypothesized that conditions in patients with CD that could potentially alter the inflammatory status of the patients, such as presence of EIM and anti-inflammatory treatment, might affect GSDMD levels. However, study results revealed no statistically significant relationship between EIM or the treatment modality of the patients and GSDMD levels.

## Discussion

This study was conceptualized and designed to investigate the role of GSDMD as a biomarker in predicting the clinical activity of CD and its relationship with the characteristics of patients with CD. The study results revealed that GSDMD levels are correlated with the severity of CD in terms of HBI, potentially identifying this novel protein as a potential biomarker in patients with CD.

Crohn’s disease is an immune-mediated, chronic, progressive disorder of the gastrointestinal tract. The disease pathogenesis is proposed to be multifactorial, but the exact underlying mechanism is unknown. The incidence and prevalence of CD are rising globally, especially in the Western world.^7^ Crohn’s disease is a progressive disorder characterized by periods of relapse and remission, which impacts the overall well-being of patients, affecting both physical and psychological aspects.^8^ Despite tremendous developments in therapeutic options for CD in the past 2 decades, response rates to medical therapy are far from satisfactory, and more than half of patients with CD require surgical therapy during the course of the disease.^9^ Crohn’s disease is also associated with extraintestinal manifestations, ranging from CD-related conditions such as arthritis and uveitis to cancers of various organs.^10^

The unpredictable nature of the disease course in CD makes it necessary for healthcare professionals to monitor disease activity and predict flares for optimal management of this disorder. Serum and fecal biomarkers, severity indices, and, more recently, deep learning models are being used and developed to assess the status and course of disease in CD.^11^^,12^ This study is one of numerous ongoing studies in the search for a biomarker of disease activity in CD, and the study results revealed a potential new protein reflecting the clinical disease course for CD: GSDMD.

Pyroptosis is an inflammation-driven cell death mechanism that can be triggered by infections or signals resulting from dangers to cellular structure and integrity. The caspase system is activated following these signals, and the GSDMD protein is cleaved into 2 parts: GSDMD-C and GSDMD-N domains. GSDMD-N is the active part of GSDMD and triggers the formation of pores in the cell membrane, leading to membrane disruption and cell death, as well as the release of intracellular components such as interleukins into the extracellular space, resulting in inflammation.^13^ Primarily a host defense mechanism, the inflammatory cascade triggered by pyroptosis can lead to an uncontrolled state of inflammation, forming the basis of and playing a role in various inflammatory-driven disorders.^14^

The roles of GSDMD and pyroptosis in the development of IBD have been investigated in various studies. Pyroptosis has been shown to play a role in IBD pathogenesis in a review by Zhang et al.^15^ In line with this finding, the burden of pyroptosis in terms of inflammasome-associated gene expressions was associated with both the disease course and response to anti-tumor necrosis factor therapy.^16^ In a study utilizing an experimental colitis model in mice, disruption of GSDMD function was found to be correlated with increased therapeutic effects.^17^ A study by Bulek and colleagues^18^ revealed that interleukin-1β (IL-1β) release secondary to GSDMD was present in mice with colitis, which was also found to be correlated with an increase in disease severity. Gasdermin D was found to play roles in 2 more aspects of disease development in a colitis model in mice, as reported in a paper by Gao et al.^19^ Gasdermin D–deficient mice were found to have a less severe disease course, and the underlying mechanisms were found to include GSDMD-induced release of IL-18 and dysregulation of gut microbiota. The culmination of all these data highlights the role of GSDMD in disease development, course, and outcome in patients with CD, which forms the basis of this study investigating serum GSDMD protein as a biomarker for assessing disease activity in patients with CD.

Gasdermin D has also been investigated and found to be associated with other disorders of the gastrointestinal tract besides IBD. As a key player in inflammation due to its role in activating the caspase system, GSDMD has been shown to be involved in cancer pathogenesis.^20^^,^^21^ The gasdermin protein family also plays a role in the pathogenesis of gastrointestinal tract disorders other than cancers, such as immune-mediated disorders and infections, although further studies are needed to fully establish their role in these conditions.^22^ Being a crucial factor in inflammation, GSDMD has been studied in inflammatory diseases of various organ systems. A study by Hu and colleagues^23^ revealed that GSDMD inhibition in acute pancreatitis led to reduced pancreatic injury, systemic inflammatory response, and organ failure in mice. Silva et al^24^ found that the severity of disease in patients with coronavirus disease 2019 was correlated with both serum and tissue levels of GSDMD. Several other studies have also investigated and revealed the role of GSDMD in the pathogenesis of neurological disorders, including acute ischemic stroke and migraine, indicating its potential as a therapeutic target in these conditons.^25^^,^^26^

Our study revealed that GSDMD is linked to clinical disease activity in CD and can be utilized as a serum biomarker to track clinical disease activity in a quick and easy manner in both outpatient and inpatient settings. In this study, the HBI was used to assess clinical disease activity, which is an easy-to-use severity index to assess disease state in CD. The Harvey–Bradshaw Index was strongly correlated with serum GSDMD levels in patients with CD, with increased levels of GSDMD being linked to more severe clinical disease states. C-reactive protein and HBI scores of the patients were also positively correlated, which is expected considering the role of CRP as an inflammatory biomarker in CD.^27^ EIMs of CD in the study population were included in the analysis, as these inflammatory conditions could potentially affect the level of an inflammatory biomarker such as GSDMD. The study results did not reveal a relationship between the presence of EIMs and GSDMD levels or HBI scores of the patients. The treatment modality of the patients at the time of enrollment and concurrent steroid usage were also not found to be correlated with GSDMD levels or HBI scores.

The major strength of this study is that, to the authors’ knowledge, it is the first study in the literature investigating the role of GSDMD as a potential biomarker in CD. The main limitation of this study is the lack of concurrent endoscopic evaluation at the time of blood sampling for GSDMD; hence, a potential correlation between endoscopic severity indices for CD and GSDMD levels could not be evaluated. In order to strengthen the role of serum GSDMD in predicting disease activity in CD, future studies should incorporate endoscopic findings of patients to obtain more data for evaluation. Endoscopic assessment of patients with CD for disease activity and biopsies of affected segments will not only provide a better understanding of the state of CD but also allow the investigation of GSDMD in mucosa through methods such as microscopy-based quantification and/or polymerase chain reaction, in addition to serum GSDMD levels.

This pioneering study revealed that serum GSDMD can be used as a biomarker to assess clinical disease activity in patients with CD. Future studies incorporating colonoscopic evaluation into the equation will provide further insight into the usability of this protein as a surrogate marker of disease progression in CD. Additionally, drug therapies targeting GSDMD may become a hot topic in the near future, hopefully strengthening the armamentarium of therapeutic options against CD.

## Figures and Tables

**Table 1.** Patient Characteristics of the Study Population

	Number of Patients (n = 61)	Percentage (%)
Sex		
Female	20	32.8
Male	41	67.2
Active smoker		
No	53	86.9
Yes	8	13.1
Treatment modality		
Conventional	35	57.4
Biologic	26	42.6
Concurrent corticosteroids		
No	58	95.1
Yes	3	4.9
Previous surgery for CD		
No	49	80.3
Yes	12	19.7
CD involvement site		
Ileum only	31	50.8
Ileocolonic involvement	30	49.2
Presence of extraintestinal Manifestations		
No	48	78.7
Yes	13	21.3
Disease state according to HBI		
Remission	30	49.2
Mild disease	18	29.5
Moderate disease	12	19.7
Severe disease	1	1.6

CD, Crohn’s disease; HBI, Harvey–Bradshaw Index.

**Table 2.** Demographic and Laboratory Data of the Study Population

	Median with Minimum and Maximum Values
Age (years)	40 (21-80)
WBC (/mm^3^)	7900 (1300-14 700)
ESR (mm/h)	12 (1-63)
CRP (mg/L)	6.0 (0.1-53.5)
GSDMD (ng/mL)	3.08 (0.88-12)
HBI score	5 (1-17)
Time since CD diagnosis (months)	49 (6-241)
	Mean ± SD
BMI (kg/m^2^)	23.0 ± 2.4

BMI, body mass index; CD, Crohn’s disease; CRP, C-reactive protein; ESR, erythrocyte sedimentation rate; GSDMD, gasdermin D; HBI, Harvey–Bradshaw Index; WBC, white blood cells.

**Table 3.** Factors Associated with Moderate and Severe Disease According to Harvey–Bradshaw Index

Variables	Coef.	OR (95% CI)	*P*
Age	0.021	1.022 (0.881-1.164)	.768
BMI	0.108	1.114 (0.446-1.783)	.751
Smoking	3.313	27.466 (23.421-31.511)	.108
CRP	0.119	1.126 (1.025-1.227)	.024
GSDMD	0.954	2.595 (1.796-3.395)	.019

Multivariate logistic regression analysis results are provided.

BMI, body mass index; CRP, C-reactive protein; GSDMD, gasdermin D, HBI, Harvey–Bradshaw Index; OR, odds ratio.

## Data Availability

The data that support the findings of this study are available on request from the corresponding author.
